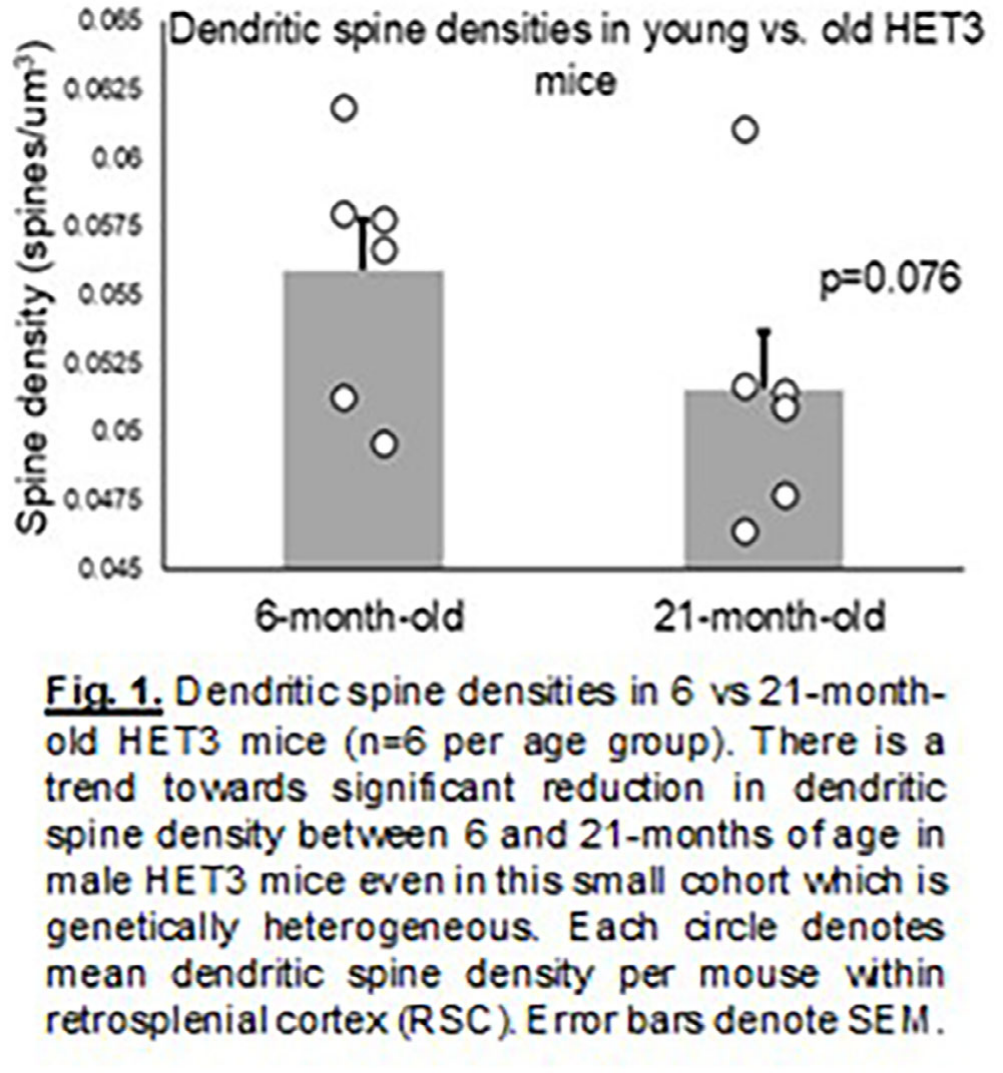# The HET3 mouse as a model of age‐dependent dendritic spine loss, proteome alterations, and cognitive decline

**DOI:** 10.1002/alz70855_103190

**Published:** 2025-12-23

**Authors:** Josh Krivinko, Susan Erickson, Akayla Lewin, Matthew L MacDonald, Jason Newman, Stacey J Sukoff Rizzo, Robert Sweet

**Affiliations:** ^1^ University of Pittsburgh, Pittsburgh, PA, USA; ^2^ University of Pittsburgh School of Medicine, Pittsburgh, PA, USA

## Abstract

**Background:**

Age‐dependent dendritic spine loss is a hypothesized mechanism by which increased chronological age lowers the threshold for developing cognitive decline after ADRD pathologies accumulate. Therefore, slowing spine loss during normal aging may be a strategy to enhance resilience to cognitive decline induced by the accumulation of ADRD pathologies. In a recent human postmortem study of precuneus tissue, we identified repurposed drugs that are predicted to reverse the proteome signature of age‐dependent spine loss, thus slowing spine loss and enhancing resilience to cognitive decline. NIA has promulgated HET3 mice as a preferred model for preclinical evaluation of anti‐aging therapies. We therefore sought to evaluate the face validity of the HET3 mouse as a model of age‐dependent spine loss and its associated proteome alterations and cognitive deficits.

**Method:**

Male HET3 mice 6 and 21 months of age (*n* = 6 per age group) were sacrificed and perfused with normal saline, after which left cerebral cortices and right hemibrains were harvested for proteomics and immunohistochemistry/confocal microscopy, respectively. Dendritic spine densities at 12‐15 randomly selected sites per mouse in retrosplenial cortex (RSC) were quantified with immunohistochemistry/confocal microscopy by colocalization of antibody‐mediated detection of spinophillin and filamentous actin by phalloidin. Left cerebral cortex gray matter was homogenized in Syn‐Per reagent and protein abundances quantified by liquid chromatography/mass spectrometry.

**Result:**

Male HET3 mice between 6 and 21 months of age exhibit reduction in spine density in RSC of large effect size (Cohen's d=0.893), nearly reaching significance in this small sample (one‐sided t‐test *p* = 0.076) (Figure 1). The magnitude of spine density reduction (7.8%) was similar to that which we previously observed between the youngest (20‐30 years of age) and oldest (>70 years of age) subjects in our study of human postmortem precuneus (8.5%). The proteome signature of aging in HET3 mice and age‐dependent cognitive deficits detected in a separate sample cohort will be presented.

**Conclusion:**

The HET3 mouse may be a viable model for studies designed to test candidate interventions for their abilities to slow age‐dependent spine loss and protect against cognitive decline. Additional studies with larger sample sizes of both sexes designed to replicate age‐dependent spine loss in HET3 are warranted.